# Supplementary material for: Degradable mesoporous semimetal antimony nanospheres for near-infrared II multimodal theranostics
Source: Nat Commun. 2022 Jan 27;13:539. doi: 10.1038/s41467-021-27835-y (PMC8795193; doi:10.1038/s41467-021-27835-y)
Supplement: Supplementary file 1 — Supplementary Information [file 41467_2021_27835_MOESM1_ESM.docx]

**Degradable Mesoporous Semimetal Antimony Nanospheres for Near-Infrared II Multimodal Theranostics**

Yu Chen,^1#^ Zhongzheng Yu,^2#^ Kai Zheng,^3^ Yaguang Ren,^4^ Meng Wang,^1^ Qiang Wu,^1^Feifan Zhou,^1^ Chengbo Liu,^4^ Liwei Liu,^1^ Jun Song,^1^* and Junle Qu^1,5^*

^#^These authors contributed equally

^1^Center for Biomedical Optics and Photonics (CBOP) & College of Physics and Optoelectronic Engineering, Key Laboratory of Optoelectronic Devices and Systems, Shenzhen University, Shenzhen 518060, P. R. China

^2^School of Chemical and Biomedical Engineering Nanyang Technological University, 637459, Singapore

^3^Northwestern Polytechnical University, School of Civil Aviation, 127 West Youyi Road, Beilin District, Xi’an; Shanxi, 710072, P.R.China

^4^Research Laboratory for Biomedical Optics and Molecular Imaging, Shenzhen Institutes of Advanced Technology, CAS Key Laboratory of Health Informatics, Chinese Academy of Sciences, Shenzhen 518055, China

^5^National Research Nuclear University MEPhI (Moscow Engineering Physics Institude), Moscow 115409, Russian Federation

E-mail: songjun@szu.edu.cn; jlqu@szu.edu.cn

**Keywords:** Mesoporous Antimony, Photodegradable, Photothermal therapy, Photoacoustic imaging


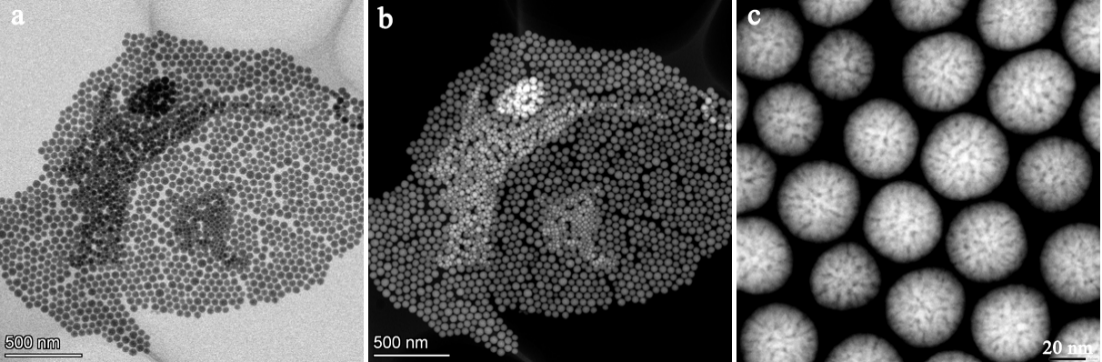


**Supplementary Figure 1**. Low-magnification (a) TEM and (b) STEM images of MSbNSs. (c) High-magnification STEM image of MSbNSs. A representative image of 3 replicates from each group is shown.


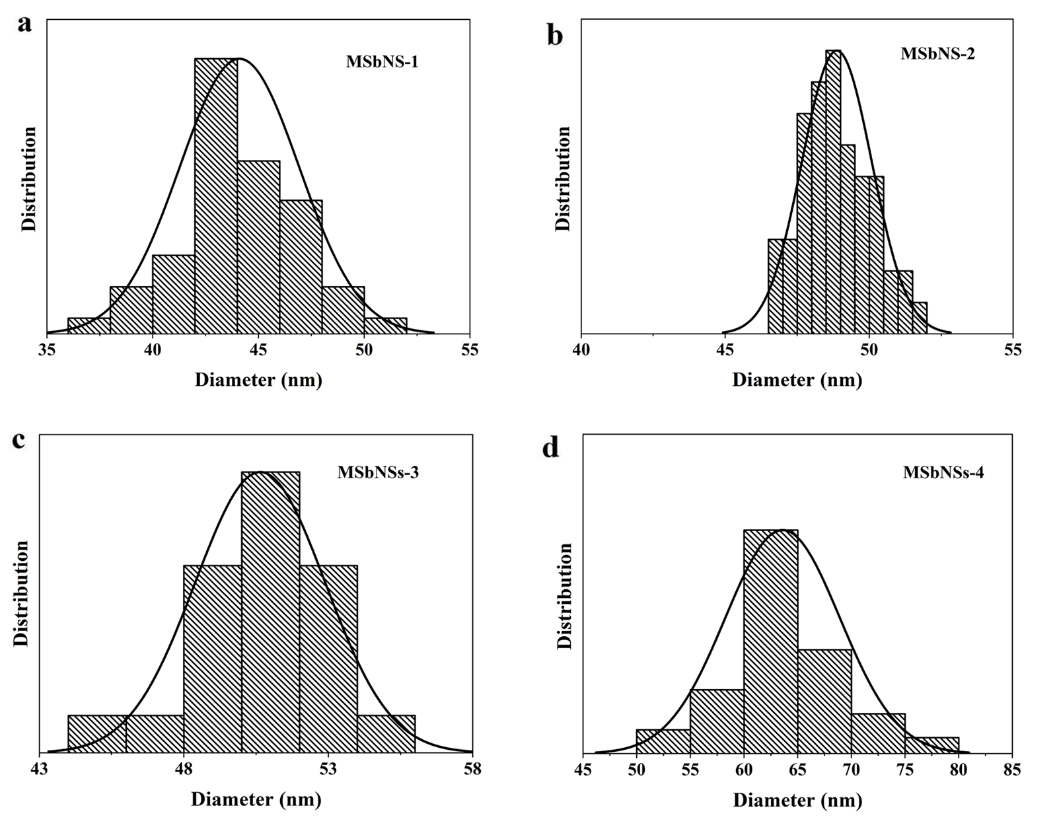


**Supplementary Figure 2**. The average diameters of (a) MSbNSs-1, (b) MSbNSs-2, (c) MSbNSs-3, (d) MSbNSs-4. Experiments were performed three times with similar results.


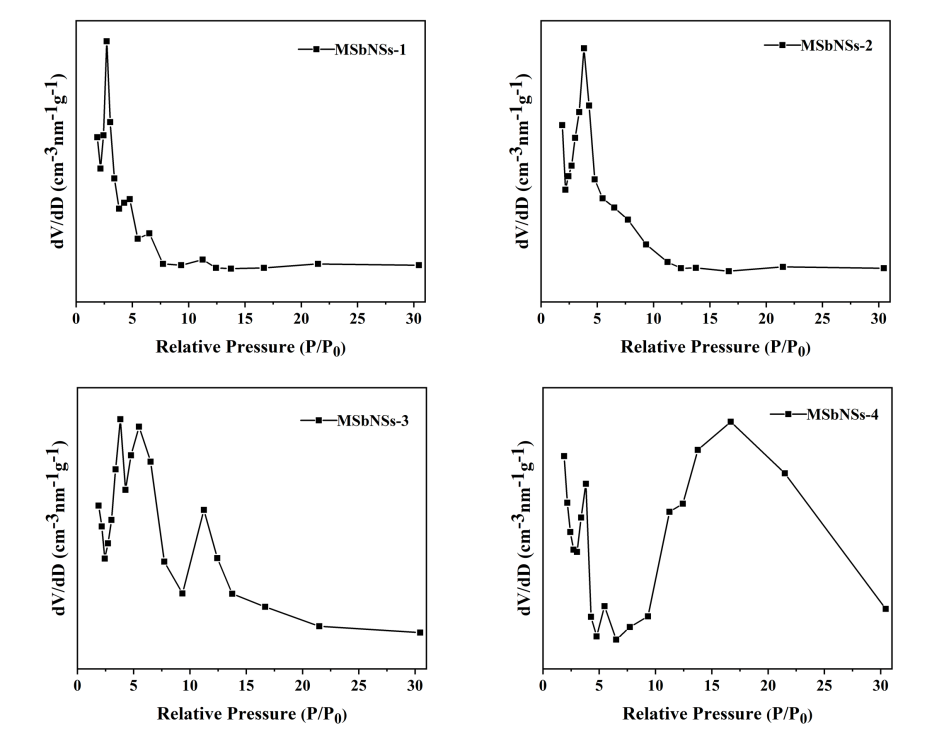


**Supplementary Figure 3**. The average pore size of MSbNSs-1/ MSbNSs-2/ MSbNSs-3/ MSbNSs-4 calculated by the BJH method. Experiments were performed three times with similar results.


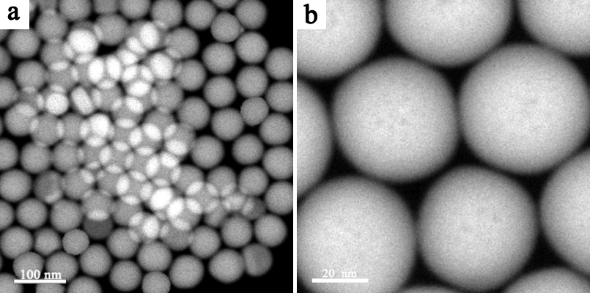


**Supplementary Figure 4**. (a) Low and (b) high-magnification STEM images of SbNSs prepared without oxygen. A representative image of 2 replicates from each group is shown


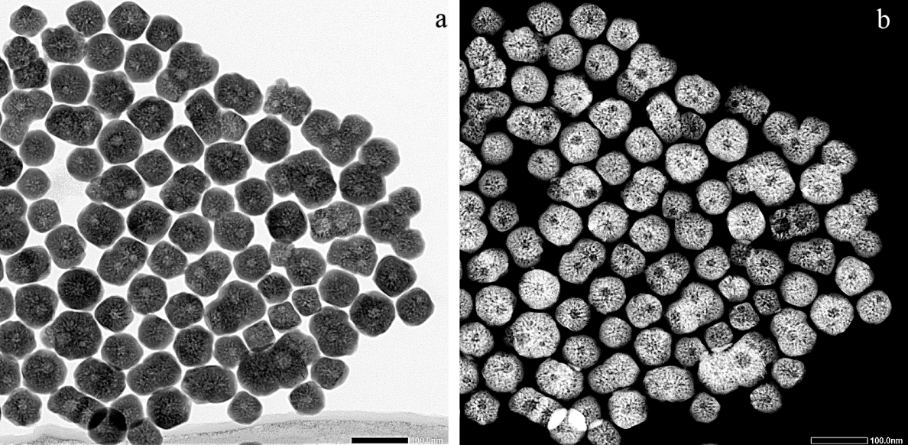


**Supplementary Figure 5**. (a) TEM and (b) STEM image of MSbNSs prepared at 190 °C and with 0.7 mL DDT, while the other experimental condition was kept constant. A representative image of 2 replicates from each group is shown.


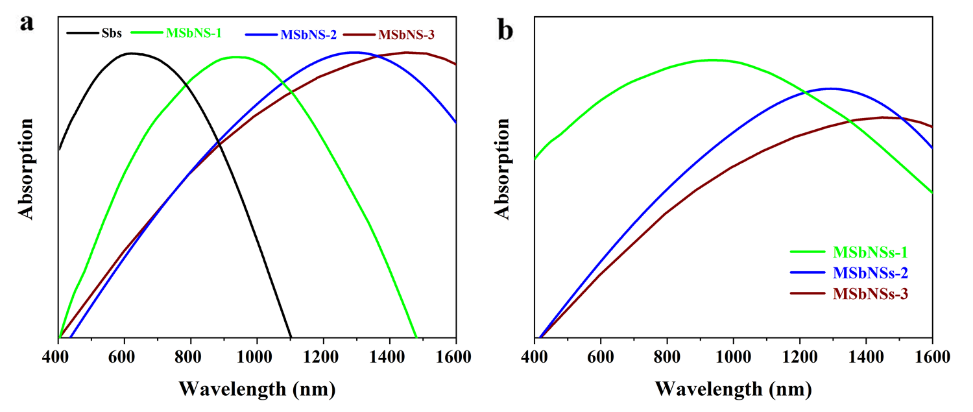


**Supplementary Figure 6**. (a) The normalized absorption spectra of Sbs/ MSbNSs-1/-2/-3, and (b) the comparison of the absorption spectra of MSbNSs-1/-2/-3 at the same concentration. Experiments were performed three times with similar results.


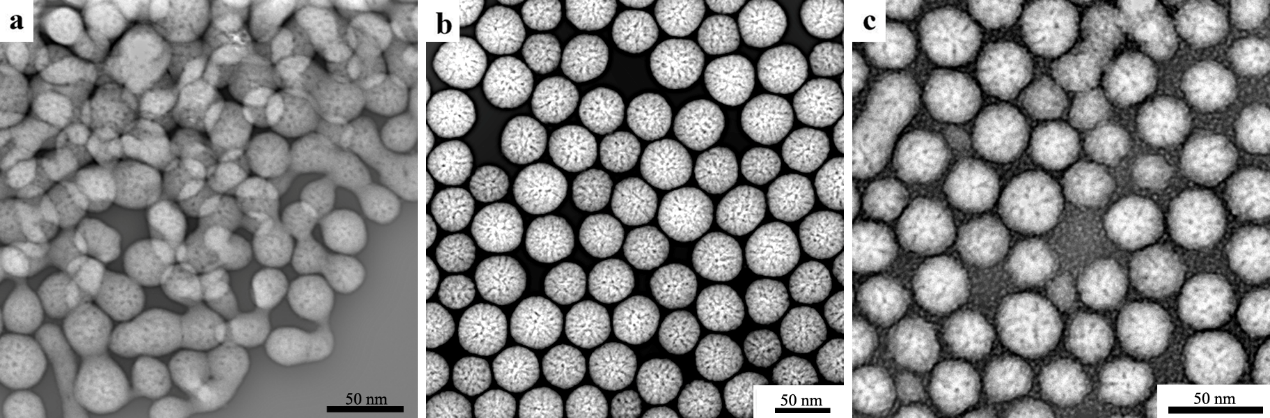


**Supplementary Figure 7**. MSbNSs fabricated with different volume of DDT ((a) 0.3 mL DDT, (b) 0.9 mL DDT, (c) 2 ml DDT) while the other experimental conditions were kept constant. A representative image of 3 replicates from each group is shown.


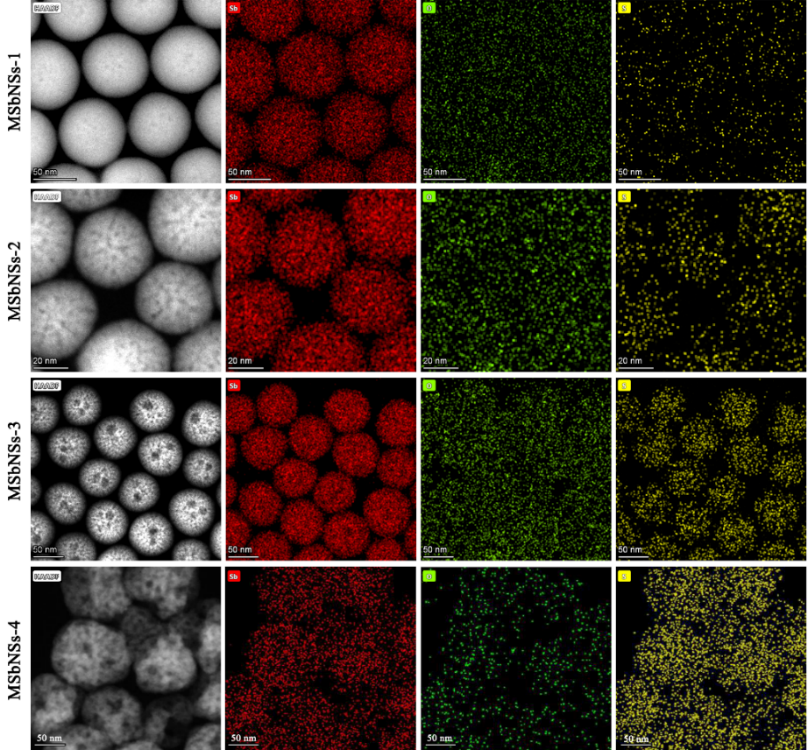


**Supplementary Figure 8**. EDS mapping of MSbNSs-1, MSbNSs-2, MSbNSs-3, MSbNSs-4, respectively. A representative image of 3 replicates from each group is shown.


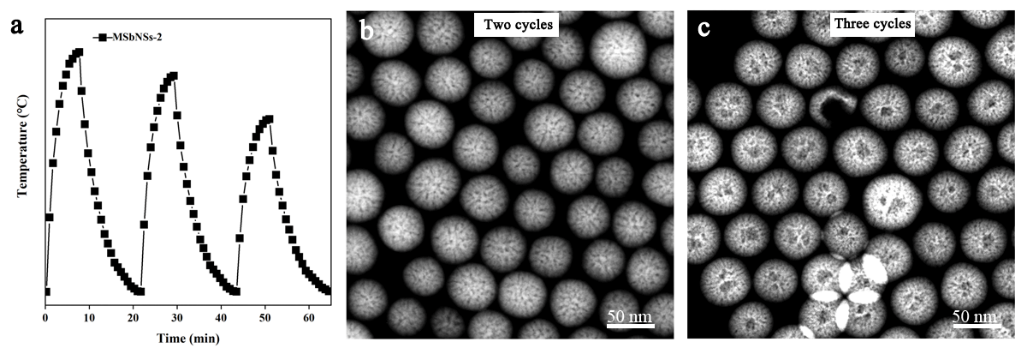


**Supplementary Figure 9**. (a) Photothermal stability of MSbNSs-2 under the irradiation of 1210 nm. (b)(c) STEM images of MSbNSs-2 before and after irradiation. A representative image of 3 replicates from each group is shown.


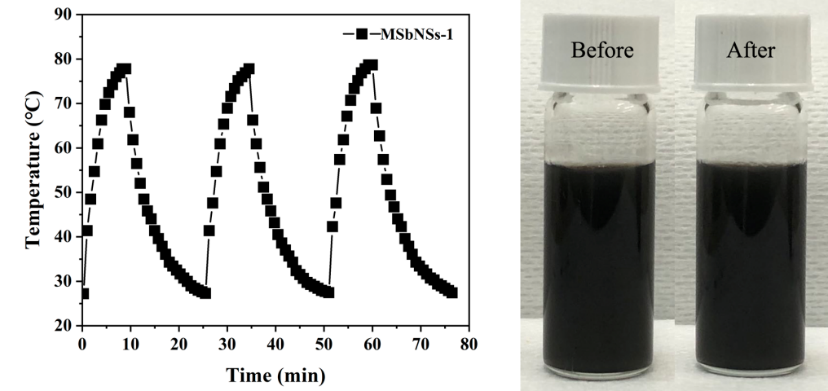


**Supplementary Figure 10**. Photothermal stability of MSbNSs-1 under the irradiation of 1210 nm. Experiments were performed three times with similar results.


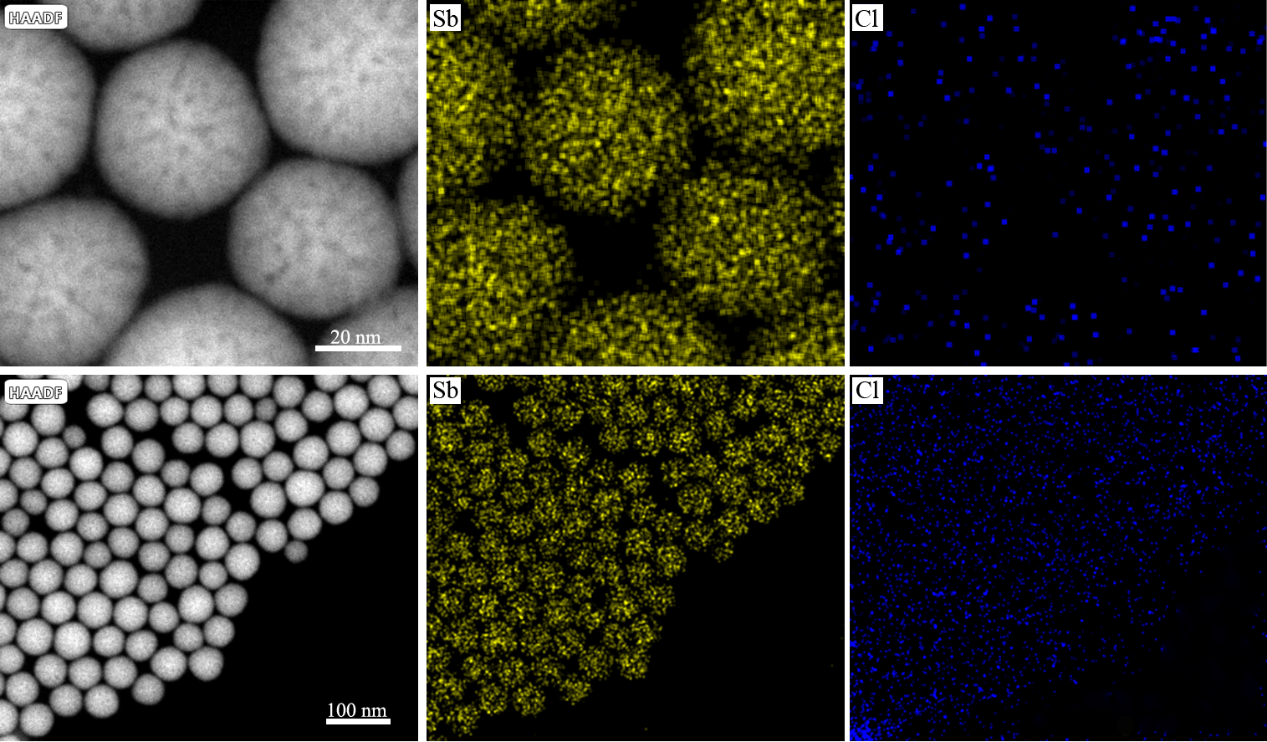


**Supplementary Figure 11**. The element mapping of Cl element in PEGylated MSbNSs-3 before and after DOX loading. A representative image of 2 replicates from each group is shown


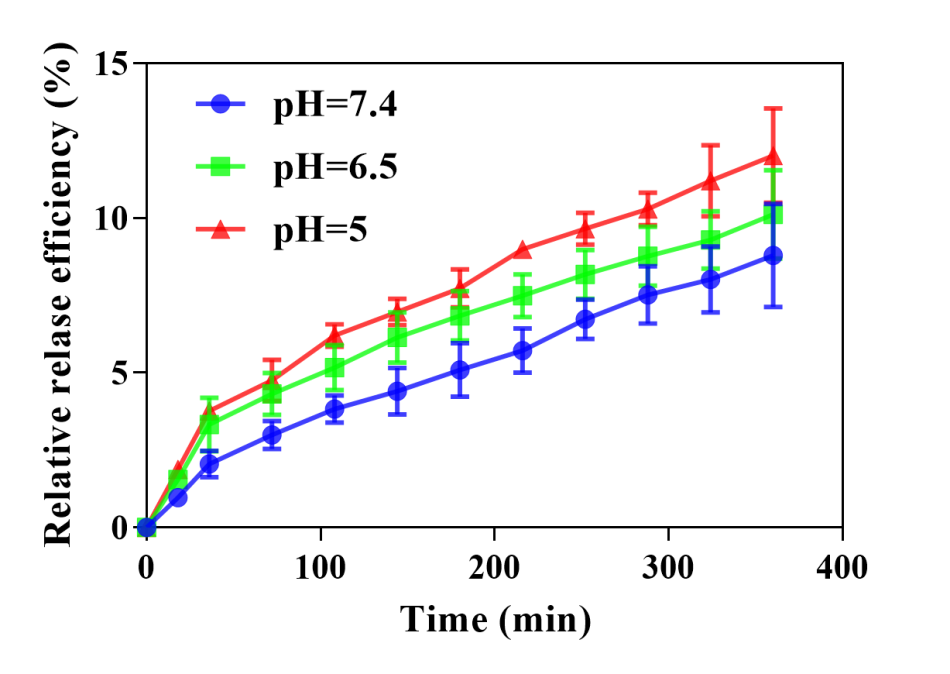


**Supplementary Figure 12**. The drug release profile at different pH values. Data are expressed as means ± SD (n = 3).

**
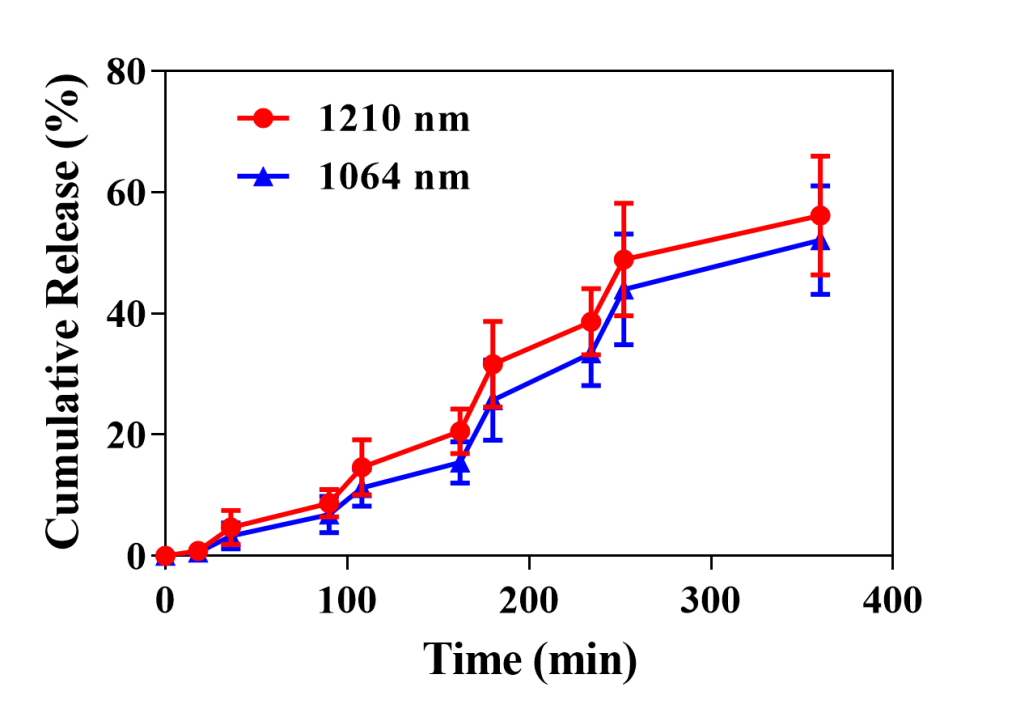
**

**Supplementary Figure 13.** On-demand drug release of PEGylated MSbNSs/DOX at different laser irradiation wavelengths. Data are expressed as means ± SD (n = 3).


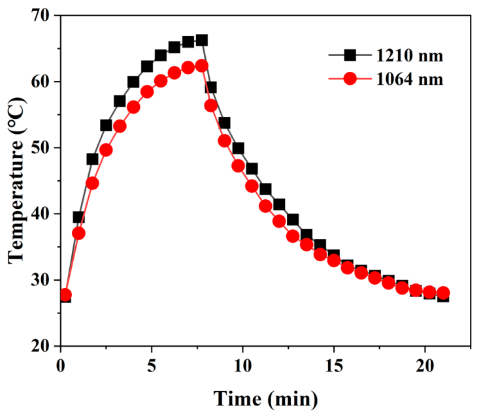


**Supplementary Figure 14**. The photothermal heating curve of the PEGylated MSbNSs-3 under laser irradiation (1210 nm/1064 nm, 1W cm^-2^) and cooling curve after turning off the laser. Experiments were performed two times with similar results.


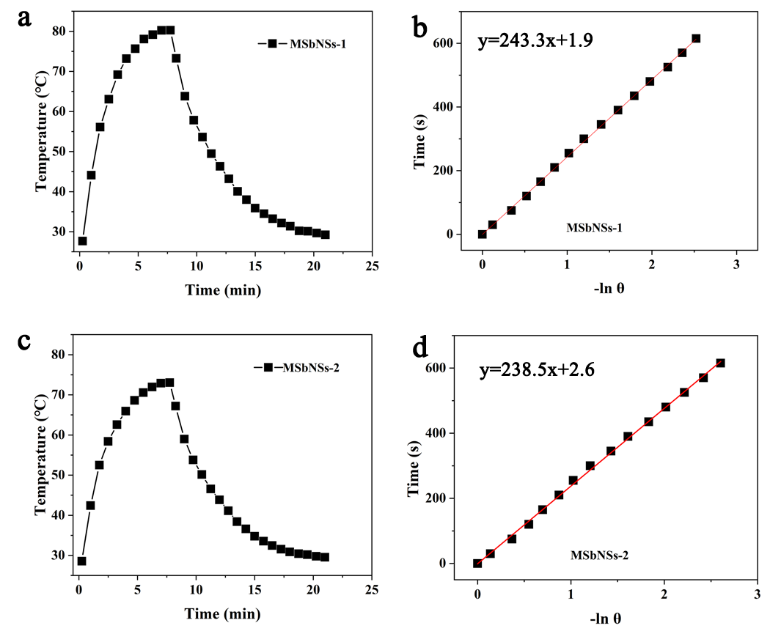


**Supplementary Figure 15**. The photothermal heating curve and the linear relationship between –lnθ and time obtained from the cooling of the PEGylated (a)(b)MSbNSs-1and (c)(d)MSbNSs-2 under laser irradiation (1210 nm, 1W cm^-2^) and cooling curve after turning off the laser. Experiments were performed two times with similar results.


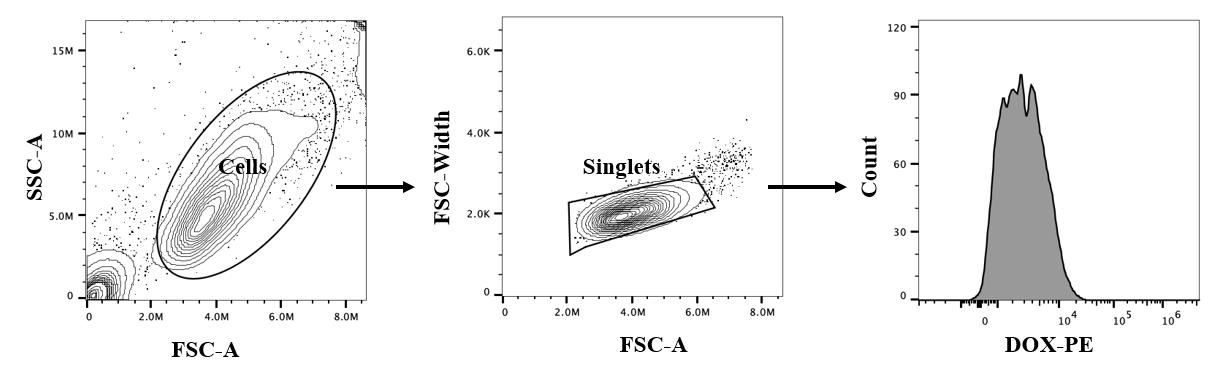


**Supplementary Figure 16**. Flow cytometry gating strategy for analysis of panc02 cells incubated PEGylated MSbNSs/DOX.


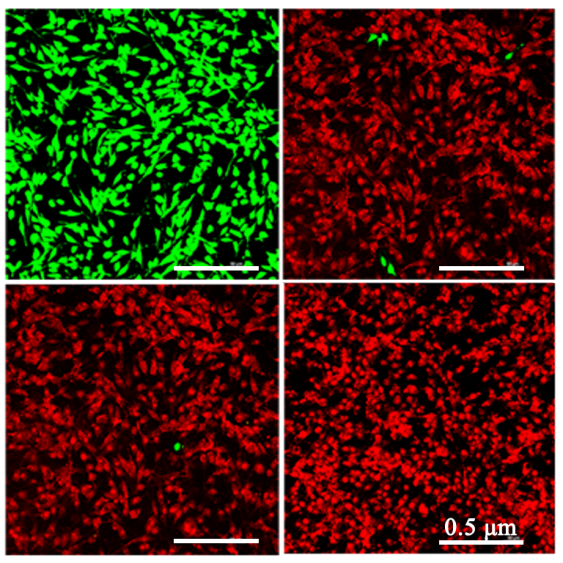


**Supplementary Figure 17**. Confocal images of calcein AM (green, live cells) and PI (red, dead cells) co-stained panc02 cells after exposed to NIR irradiation (1210 nm, 1 W cm^-2^). A representative image of 3 replicates from each group is shown.


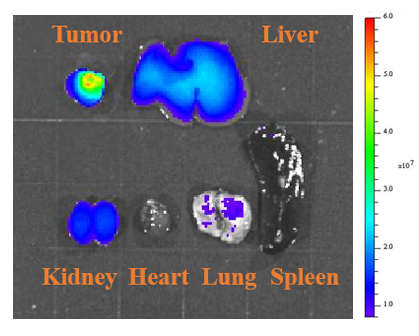


**Supplementary Figure 18**. The accumulation of PEGylated MSbNSs in tumor and major organs at 8h post-injection. A representative image of two individual mice per group is shown.


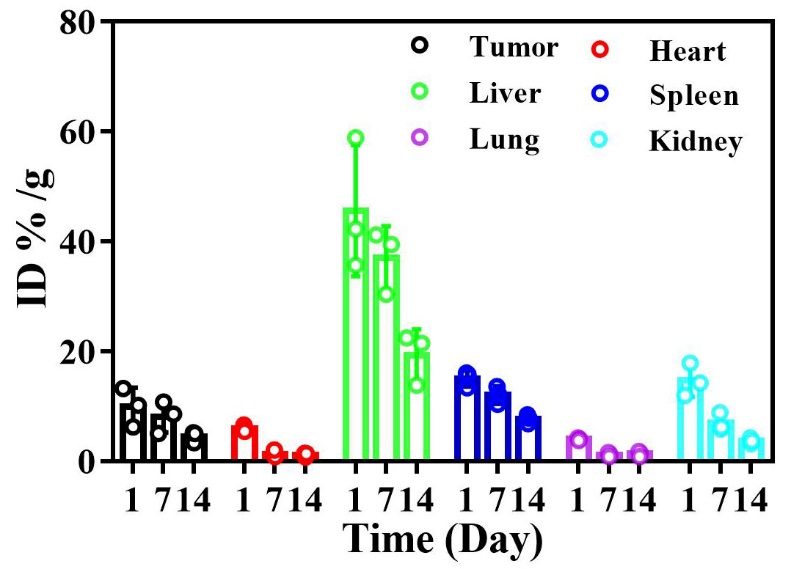


**Supplementary Figure 19.** The time-dependent biodistribution of PEGylated MSbNSs without irradiation. After i. v. injection of PEGylated MSbNSs (50 mg/kg), the tumor and major organs of mice (including heart, liver, spleen, lung and kidney) were collected at the 1st, 7th, and 14th day for ICP-MS measurement of Sb levels to determine the biodistribution of nanoparticles. Data are expressed as means ± SD (n = 3).


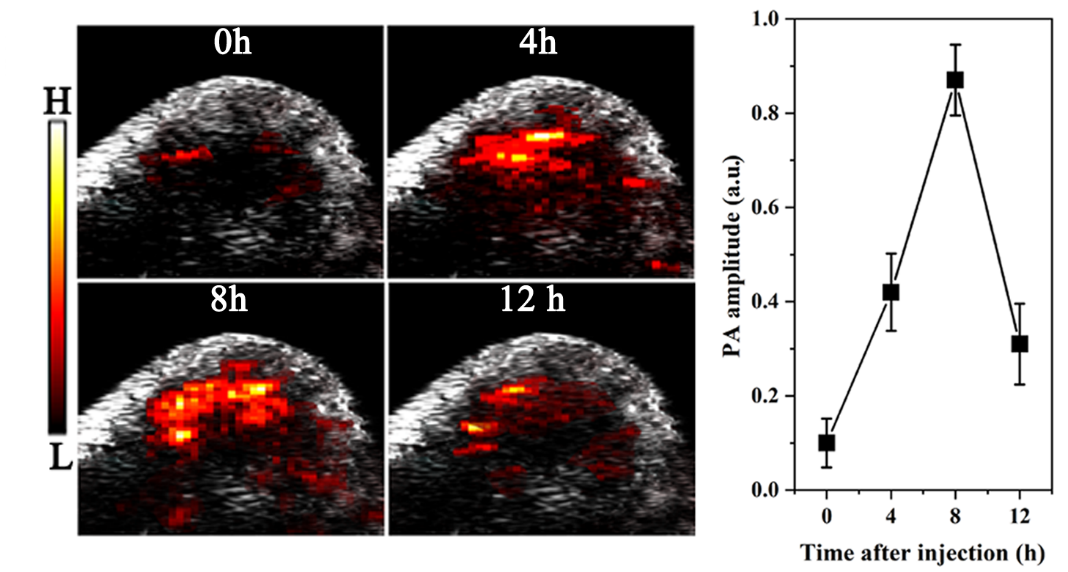


**Supplementary Figure 20**. The change of PA intensity over time with PEGylated MSbNSs-3/DOX injected at the tumor site. Data are expressed as means ± SD (n = 3).

.


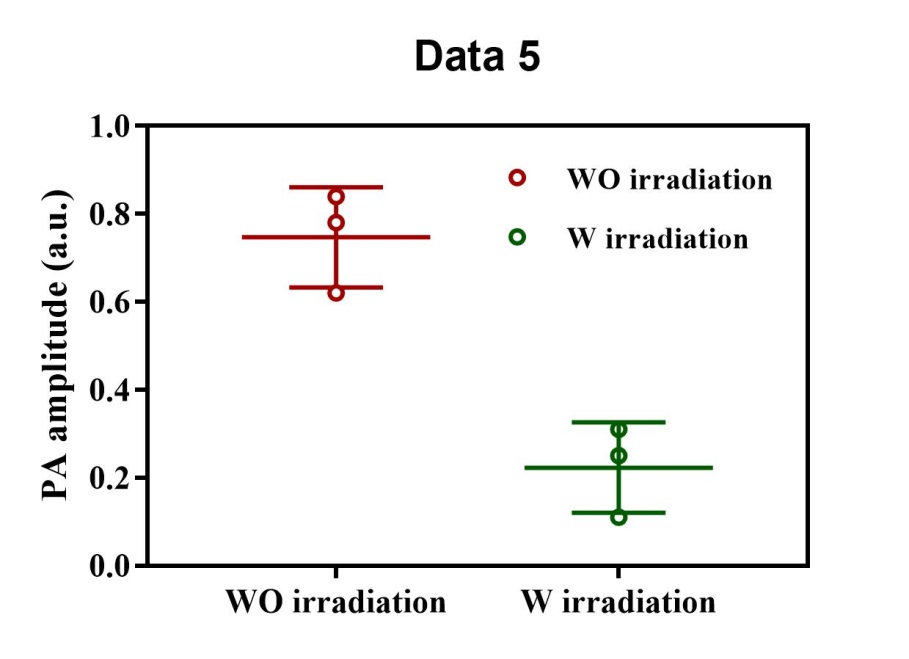


**Supplementary Figure 21**. The change of the PA intensity with and without laser irradiation. Data are expressed as means ± SD (n = 3).


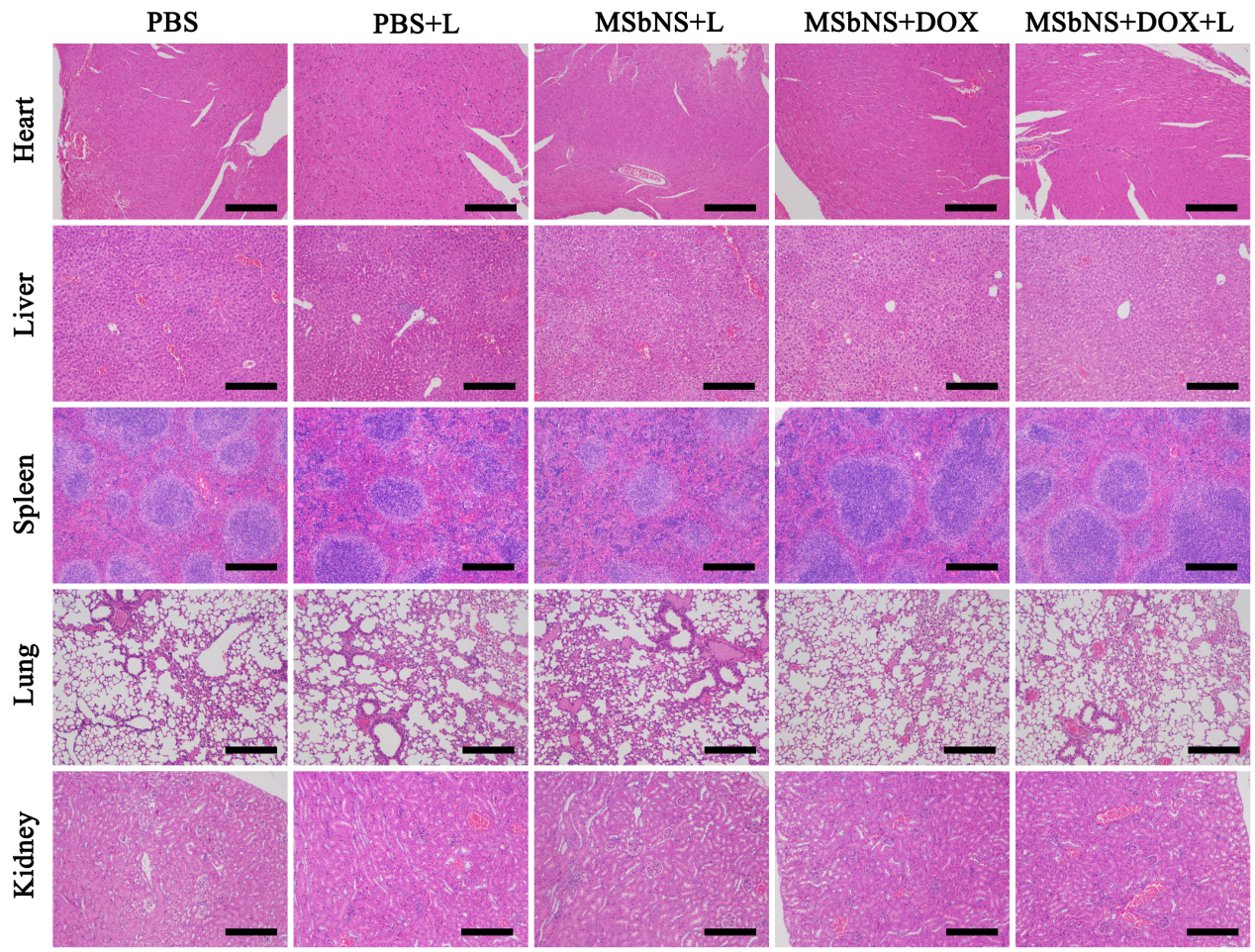


**Supplementary Figure 22**. Representative H&E-stained images of major organs including heart, liver, spleen, lung and kidney of mice receiving different treatments. Scale bar=100μm. A representative image of 2 individual mice per group is shown is shown.


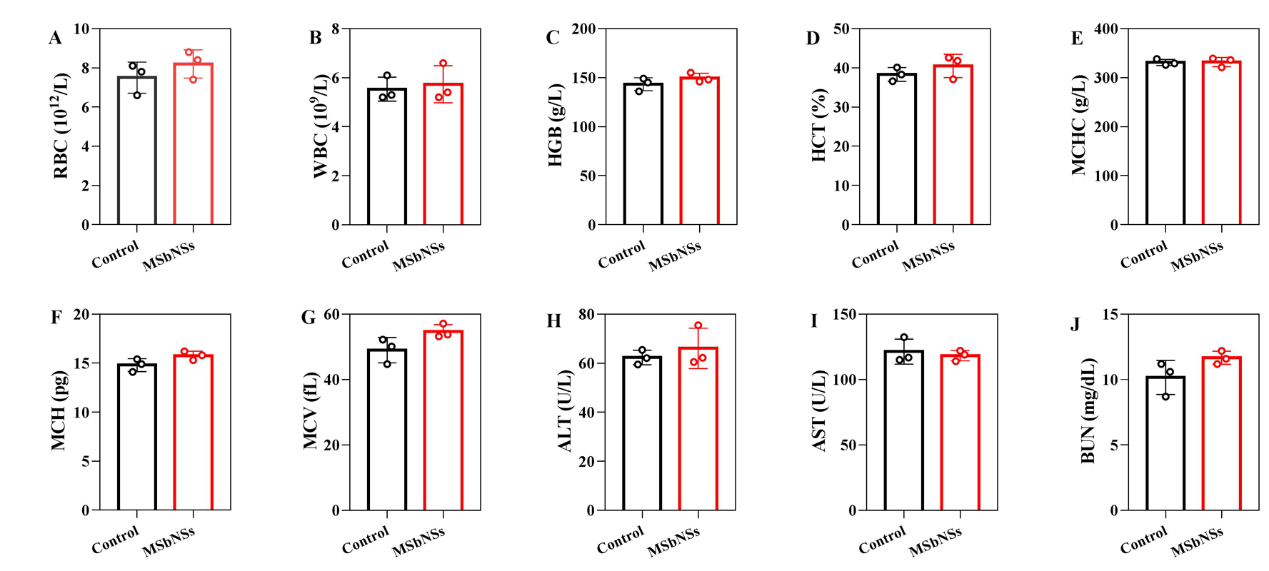


**Supplementary Figure 23.** Hematology and blood biochemistry analysis of mice *i. v.* injected with PEGylated MSbNSs (50 mg/kg) at 14^th^ day. The examined parameters included (A) Red blood cell (RBC) counts; (B) White blood cell (WBC) counts; (C) Hemoglobin (HGB); (D) Hematocrit (HCT); (E) Mean corpuscaular haemoglobin concentration (MCHC); (F) Mean corpuscular hemoglobin (MCH); (G) Mean corpuscular volume (MCV); (H) Aspartate aminotransferase (AST); (I) Alanine aminotransferase (ALT); and (J) Blood urea nitrogen (BUN). Data are expressed as means ± SD (n = 3).


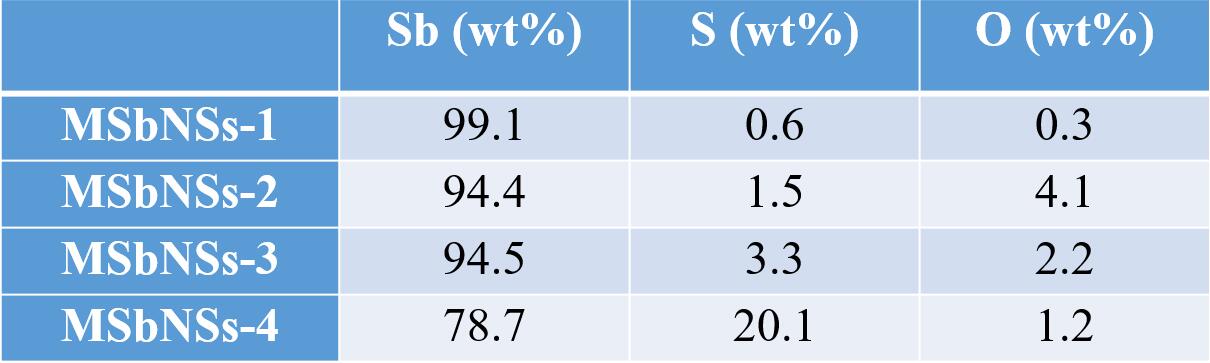


**Supplementary Table 1**. The mass percentages of Sb, O, and S elements in MSbNSs-1, MSbNSs-2, MSbNSs-3, MSbNSs-4, respectively. A representative data of 2 replicates from each group is shown.
