# Supplementary material for: Degradable mesoporous semimetal antimony nanospheres for near-infrared II multimodal theranostics
Source: Nat Commun. 2022 Jan 27;13:539. doi: 10.1038/s41467-021-27835-y (PMC8795193; doi:10.1038/s41467-021-27835-y)
Supplement: Supplementary file 2 — Reporting Summary [file 41467_2021_27835_MOESM2_ESM.pdf]

nature portfolio

Corresponding author(s): Jule Qu
Last updated by author(s): Sep 25, 2021

Reporting Summary

Nature Portfolio wishes to improve the reproducibility of the work that we publish. This form provides structure for consistency and transparency in reporting. For further information on Nature Portfolio policies, see our Editorial Policies and the Editorial Policy Checklist.

Statistics

For all statistical analyses, confirm that the following items are present in the figure legend, table legend, main text, or Methods section.

n/a	Confirmed	
☐	☒	The exact sample size (n) for each experimental group/condition, given as a discrete number and unit of measurement
☐	☒	A statement on whether measurements were taken from distinct samples or whether the same sample was measured repeatedly
☐	☒	The statistical test(s) used AND whether they are one- or two-sided<br>*Only common tests should be described solely by name; describe more complex techniques in the Methods section.*
☐	☒	A description of all covariates tested
☐	☒	A description of any assumptions or corrections, such as tests of normality and adjustment for multiple comparisons
☐	☒	A full description of the statistical parameters including central tendency (e.g. means) or other basic estimates (e.g. regression coefficient) AND variation (e.g. standard deviation) or associated estimates of uncertainty (e.g. confidence intervals)
☐	☒	For null hypothesis testing, the test statistic (e.g. F, t, r) with confidence intervals, effect sizes, degrees of freedom and P value noted<br>*Give P values as exact values whenever suitable.*
☒	☐	For Bayesian analysis, information on the choice of priors and Markov chain Monte Carlo settings
☒	☐	For hierarchical and complex designs, identification of the appropriate level for tests and full reporting of outcomes
☒	☐	Estimates of effect sizes (e.g. Cohen's d, Pearson's r), indicating how they were calculated

Our web collection on statistics for biologists contains articles on many of the points above.

Software and code

Policy information about availability of computer code

Data collection	No software was used for data collection.
Data analysis	The statistical analysis of data was performed on Microsoft Excel 2010 and Origin 9.1. The finite-difference-time-domain (FDTD) simulation was obtained on 8.11.337 version, Lumerical Solutions, Inc.

For manuscripts utilizing custom algorithms or software that are central to the research but not yet described in published literature, software must be made available to editors and reviewers. We strongly encourage code deposition in a community repository (e.g. GitHub). See the Nature Portfolio guidelines for submitting code & software for further information.

Data

Policy information about availability of data

All manuscripts must include a data availability statement. This statement should provide the following information, where applicable:

- Accession codes, unique identifiers, or web links for publicly available datasets
- A description of any restrictions on data availability
- For clinical datasets or third party data, please ensure that the statement adheres to our policy

Source data are provided with this paper. The data supporting our results within this article and Supplementary Information are available from the corresponding author upon request.

Field-specific reporting

Please select the one below that is the best fit for your research. If you are not sure, read the appropriate sections before making your selection.

- [x] Life sciences
- [ ] Behavioural & social sciences
- [ ] Ecological, evolutionary & environmental sciences

For a reference copy of the document with all sections, see nature.com/documents/nr-reporting-summary-flat.pdf

Life sciences study design

All studies must disclose on these points even when the disclosure is negative.

Sample size	We have not used a statistical method to decide the sample size. The group sizes (at least n>3 per group) represents the minimum number animals needed to reach statistical significance ($p < 0.05$) between experimental groups.
Data exclusions	No data were excluded in this study.
Replication	Experiments were repeated at least 3 independent experiments with similar results to ensure the correct conclusion.
Randomization	Mice were randomly allocated into experimental groups.
Blinding	We were blinded to group allocation during experiments.

Reporting for specific materials, systems and methods

We require information from authors about some types of materials, experimental systems and methods used in many studies. Here, indicate whether each material, system or method listed is relevant to your study. If you are not sure if a list item applies to your research, read the appropriate section before selecting a response.

Materials & experimental systems

n/a	Involved in the study	
x		Antibodies
	x	Eukaryotic cell lines
x		Palaeontology and archaeology
	x	Animals and other organisms
x		Human research participants
x		Clinical data
x		Dual use research of concern

Methods

n/a	Involved in the study	
x		ChIP-seq
	x	Flow cytometry
x		MRI-based neuroimaging

Eukaryotic cell lines

Policy information about cell lines

Cell line source(s)	panc 02 cells purchased from American Tissue Culture Collection (ATCC).
Authentication	Identity of the cell lines were frequently checked by their morphological features but have not been authenticated by the short tandem repeat (STR) profiling.
Mycoplasma contamination	No contamination was detected by the supplier.
Commonly misidentified lines (See ICLAC register)	These cell lines that we used were not listed in commonly misidentified lines in ICLAC Register.

Animals and other organisms

Policy information about studies involving animals; ARRIVE guidelines recommended for reporting animal research

Laboratory animals	All of our animal experiments were conducted in compliance with the guidelines established by the Animal Ethical and Welfare Committee of Shenzhen University (AEWC-SZU). Female C57BL/6 mice (6-8 weeks) were used.
Wild animals	The study did not involve wild animals.
Field-collected samples	The study did not involve samples collected from the field.
Ethics oversight	No ethical issues.

Note that full information on the approval of the study protocol must also be provided in the manuscript.

Flow Cytometry

Plots

Confirm that:

- [ ] The axis labels state the marker and fluorochrome used (e.g. CD4-FITC).
- [x] The axis scales are clearly visible. Include numbers along axes only for bottom left plot of group (a 'group' is an analysis of identical markers).
- [x] All plots are contour plots with outliers or pseudocolor plots.
- [x] A numerical value for number of cells or percentage (with statistics) is provided.

Methodology

Sample preparation	Only one sample to prove the cellular uptake in this study. For flow-cytometry analysis, panc02 cancer cells were seeded into 6-well plates (5 × 105) and incubated overnight at 37 °C. Then, MSbNSs-3/DOX (≈1 × 106) dispersed in Dulbecco's modified Eagles's medium were added and incubated at 37 °C. At specified intervals, the cells were washed with phosphate-buffered saline (PBS) three times, harvested by trypsinization, centrifuged at 3000 rpm for 3 min, resuspended in PBS and analyzed using the Beckman CytoFLEX flow-cytometry system. The percentages of cells interacting with panc02 were determined by signal of DOX.
Instrument	Beckman CytoFLEX flow-cytometry system
Software	Cytomics FC 500
Cell population abundance	No sorting was performed.
Gating strategy	No gating strategy was applied in this study.

- [x] Tick this box to confirm that a figure exemplifying the gating strategy is provided in the Supplementary Information.
